# Supplementary material for: Dental complexity and diet in amniotes: A meta-analysis
Source: PLoS One. 2024 Feb 2;19(2):e0292358. doi: 10.1371/journal.pone.0292358 (PMC10836679; doi:10.1371/journal.pone.0292358)
Supplement: S1 Table — Asterisks (*) indicate significant difference from 0. (PDF) [file pone.0292358.s002.pdf]

	Lowers	Uppers
Fixed-effects model, not phylogenetically corrected	0.97*	0.94*
Fixed-effects model, phylogenetically corrected	-8.08	-7.14
Mixed-effects model	-13.18	-7.83
