# Supplementary material for: Dental complexity and diet in amniotes: A meta-analysis
Source: PLoS One. 2024 Feb 2;19(2):e0292358. doi: 10.1371/journal.pone.0292358 (PMC10836679; doi:10.1371/journal.pone.0292358)
Supplement: S2 Table — (PDF) [file pone.0292358.s003.pdf]

| Author | Year | Sample within study | Study group | Herbivore diet | Faunivore diet | SMD |
|---|---|---|---|---|---|---|
| Berthaume et al. | 2019 b | 1 | Prosimians | Folivore | Insectivore | -0.25 |
| Berthaume et al. | 2019 b | 2 | Prosimians | Frugivore | Insectivore | -0.05 |
| Bunn et al. | 2011 | 1 | Primates | Folivore | Insectivore | -1.34 |
| Bunn et al. | 2011 | 2 | Euarchontans | Frugivore | Insectivore | -7.45 |
| Christensen and Melstrom | 2021 | 1 | Squamates | Herbivore | Carnivore | 1.77 |
| Christensen and Melstrom | 2021 | 2 | Squamates | Herbivore | Insectivore | 1.05 |
| Evans et al. | 2007 | 1 | Carnivorans | Herbivore | Carnivore | 3.21 |
| Evans et al. | 2007 | 2 | Muroid rodents | Herbivore | Carnivore | 2.71 |
| Fulwood | 2019 | 1 | Strepsirrhine primates | Folivore | Insectivore | 0.45 |
| Fulwood | 2019 | 2 | Strepsirrhine primates | Frugivore | Insectivore | -0.32 |
| López-Aguirre et al. | 2021 | 1 | Noctilionoid bats | Frugivore | Carnivore-Piscivore | 0.56 |
| López-Aguirre et al. | 2021 | 2 | Noctilionoid bats | Frugivore | Insectivore | 0.05 |
| Melstrom | 2016 | 1 | Dentigerous saurians | Herbivore | Carnivore | 1.71 |
| Melstrom | 2016 | 2 | Dentigerous saurians | Herbivore | Insectivore | 0.91 |
| Pérez-Ramos et al. | 2020 | | Bears | Folivore-frugivore | Faunivore | 0.48 |
| Pineda-Munoz et al. | 2016 | 1 | Terrestrial mammals | Frugivore | Carnivore | 3.47 |
| Pineda-Munoz et al. | 2016 | 2 | Terrestrial mammals | Frugivore | Insectivore | 1.03 |
| Pineda-Munoz et al. | 2016 | 3 | Terrestrial mammals | Granivore | Carnivore | 2.81 |
| Pineda-Munoz et al. | 2016 | 4 | Terrestrial mammals | Granivore | Insectivore | 0.64 |
| Pineda-Munoz et al. | 2016 | 5 | Terrestrial mammals | Herbivore | Carnivore | 3.07 |
| Pineda-Munoz et al. | 2016 | 6 | Terrestrial mammals | Herbivore | Insectivore | 0.87 |
| Santana et al. | 2011 | | Phyllostomid bats | Frugivore | Insectivore | 2.72 |
| Selig et al. | 2021 | 1 | Euarchontans | Folivore | Insectivore | 1.64 |
| Selig et al. | 2021 | 2 | Euarchontans | Frugivore | Insectivore | -0.78 |

| | | | | | | |
|---|---|---|---|---|---|---|
| Selig et al. | 2020 | 1 | Euarchontans | Folivore | Insectivore | 1.92 |
| Selig et al. | 2020 | 2 | Euarchontans | Frugivore | Insectivore | 0.29 |
| Smith | 2017 | 1 | Terrestrial mammals | Folivore | Insectivore | -2.80 |
| Smith | 2017 | 2 | Terrestrial mammals | Folivore | Carnivore | -0.37 |
| Smith | 2017 | 3 | Terrestrial mammals | Frugivore | Insectivore | 7.02 |
| Smith | 2017 | 4 | Terrestrial mammals | Frugivore | Carnivore | 17.12 |
| Spradley | 2017 | 1 | Marsupials | Folivore | Faunivore | 1.88 |
| Spradley | 2017 | 2 | Marsupials | Folivore | Insectivore | 1.28 |
| Spradley | 2017 | 3 | Marsupials | Frugivore | Insectivore | -0.45 |
| Spradley | 2017 | 4 | Marsupials | Frugivore | Faunivore | 0.80 |
| Tiphaine et al. | 2013 | 1 | Muroid rodents | Frugivore-granivore | Piscivore | 1.44 |
| Tiphaine et al. | 2013 | 2 | Muroid rodents | Frugivore-granivore | Insectivore | 2.11 |
| Tiphaine et al. | 2013 | 3 | Muroid rodents | Folivore | Piscivore | 3.74 |
| Tiphaine et al. | 2013 | 4 | Muroid rodents | Folivore | Insectivore | 7.55 |
| Ungar et al. | 2016 | 1 | Platyrrhine primates | Folivore-frugivore | Insectivore-gumivore | 1.31 |
| Ungar et al. | 2016 | 2 | Platyrrhine primates | Frugivore-granivore | Insectivore-gumivore | 1.27 |
| Ungar et al. | 2016 | 3 | Platyrrhine primates | Frugivore-folivore | Insectivore-gumivore | 2.73 |
| Ungar et al. | 2016 | 4 | Platyrrhine primates | Hard-object frugivore | Insectivore-gumivore | 1.77 |
| Waldman et al. | 2023 | | Carnivorans | Herbivore | Carnivore | -1.00 |

| Herbivore N | Herbivore mean | Herbivore SD | Faunivore N | Faunivore mean | Faunivore SD | Teeth | Upper/ Lower | OPC/OPCR | Cropping method |
|---|---|---|---|---|---|---|---|---|---|
| 7 | 81.9814815 | 13.0680405 | 9 | 85.7314815 | 14.7939658 | Single | Lower | OPCR | EEC |
| 4 | 84.9411765 | 12.9265592 | 9 | 85.7314815 | 14.7939658 | Single | Lower | OPCR | EEC |
| 6 | 51.698 | 1.53 | 5 | 53.646 | 1.014 | Single | Lower | OPCR | EEC |
| 4 | 43.15 | 1.512 | 5 | 53.646 | 1.014 | Single | Lower | OPCR | EEC |
| 6 | 10.8128484 | 2.95271845 | 3 | 5.3998 | 2.01103587 | Row | Lower | OPCR | Crown above gumline |
| 6 | 10.8128484 | 2.95271845 | 5 | 7.94923 | 1.77317085 | Row | Lower | OPCR | Crown above gumline |
| 2 | 226 | 43.8406204 | 24 | 82.75 | 43.2266524 | Row | Lower | OPC | 2.5D |
| 5 | 237 | 33.5633729 | 6 | 156.333333 | 20.8198623 | Row | Lower | OPC | 2.5D |
| 15 | 103.59 | 28.58 | 7 | 91.83 | 15.14 | Single | Lower | OPC | EEC |
| 19 | 87.35 | 12.98 | 7 | 91.83 | 15.14 | Single | Lower | OPCR | EEC |
| 4 | 102.935 | 46.3350008 | 5 | 82.524 | 14.2179158 | Single | Lower | OPCR | BCO |
| 4 | 102.935 | 46.3350008 | 5 | 100.3 | 45.7396371 | Single | Lower | OPCR | BCO |
| 15 | 10.4463977 | 2.91785407 | 13 | 5.44778302 | 2.74702862 | Row | Lower | OPCR | Crown above gumline |
| 15 | 10.4463977 | 2.91785407 | 24 | 8.34291954 | 1.75569065 | Row | Lower | OPCR | Crown above gumline |
| 3 | 223.02 | 113.653627 | 3 | 174.286667 | 11.0736459 | Row | Upper | OPCR | EEC |
| 13 | 218.307692 | 38.9323872 | 23 | 66 | 44.9693352 | Row | Lower | OPCR | 2.5D |
| 13 | 218.307692 | 38.9323872 | 21 | 176.47619 | 39.829159 | Row | Lower | OPCR | 2.5D |
| 5 | 206.2 | 64.6351298 | 23 | 66 | 44.9693352 | Row | Lower | OPCR | 2.5D |
| 5 | 206.2 | 64.6351298 | 21 | 176.47619 | 39.829159 | Row | Lower | OPCR | 2.5D |
| 48 | 218.333333 | 50.7967721 | 23 | 66 | 44.9693352 | Row | Lower | OPCR | 2.5D |
| 48 | 218.333333 | 50.7967721 | 21 | 176.47619 | 39.829159 | Row | Lower | OPCR | 2.5D |
| 6 | 424.666667 | 82.4564532 | 7 | 218.542857 | 58.562612 | Row | Lower | OPC | 2.5D |
| 4 | 81.201 | 4.46451 | 15 | 66.9625 | 8.89651 | Single | Lower | OPCR | EEC |
| 3 | 60.0556 | 4.383 | 15 | 66.9625 | 8.89651 | Single | Lower | OPCR | EEC |

| | | | | | | | | | | |
|---|---|---|---|---|---|---|---|---|---|---|
| 5 | 76.3722222 | 3.32251427 | | 6 | 67.0150463 | 5.16771647 | Row | Lower | OPCR | EEC |
| 7 | 70.0565476 | 12.4387197 | | 6 | 67.0150463 | 5.16771647 | Row | Lower | OPCR | EEC |
| 5 | 66.8 | 13.4756966 | | 3 | 106.391111 | 9.50344635 | Single | Lower | OPCR | EEC |
| 5 | 66.8 | 13.4756966 | | 3 | 71.6666667 | 5.37380064 | Single | Lower | OPCR | EEC |
| 2 | 183.543333 | 3.05941534 | | 3 | 106.391111 | 9.50344635 | Single | Lower | OPCR | EEC |
| 2 | 183.543333 | 3.05941534 | | 3 | 71.6666667 | 5.37380064 | Single | Lower | OPCR | EEC |
| 11 | 78.5454546 | 10.4465653 | | 2 | 54.65 | 21.0010714 | Single | Lower | OPCR | EEC |
| 11 | 78.5454546 | 10.4465653 | | 15 | 66.64 | 7.83597929 | Single | Lower | OPCR | EEC |
| 14 | 62.9142857 | 8.32658921 | | 15 | 66.64 | 7.83597929 | Single | Lower | OPCR | EEC |
| 14 | 62.9142857 | 8.32658921 | | 2 | 54.65 | 21.0010714 | Single | Lower | OPCR | EEC |
| 5 | 201.7 | 55.3608616 | | 2 | 116 | 14.1421356 | Single | Upper | OPCR | EEC |
| 5 | 201.7 | 55.3608616 | | 4 | 101.625 | 7.65261393 | Single | Upper | OPCR | EEC |
| 3 | 166 | 6.38357267 | | 2 | 116 | 14.1421356 | Single | Upper | OPCR | EEC |
| 3 | 166 | 6.38357267 | | 4 | 101.625 | 7.65261393 | Single | Upper | OPCR | EEC |
| 2 | 53.563 | 11.289 | | 2 | 33.469 | 4.747 | Single | Upper | OPCR | BCO |
| 2 | 45.029 | 5.5 | | 2 | 33.469 | 4.747 | Single | Upper | OPCR | BCO |
| 2 | 64.972 | 7.89 | | 2 | 33.469 | 4.747 | Single | Upper | OPCR | BCO |
| 4 | 61.75 | 14.457 | | 2 | 33.469 | 4.747 | Single | Upper | OPCR | BCO |
| 3 | 70.0833333 | 5.46913689 | | 27 | 116.185185 | 46.3228944 | Row | Lower | OPCR | EEC |
