# Supplementary figures and images for: Dental complexity and diet in amniotes: A meta-analysis
Source: PLoS One. 2024 Feb 2;19(2):e0292358. doi: 10.1371/journal.pone.0292358 (PMC10836679; doi:10.1371/journal.pone.0292358)

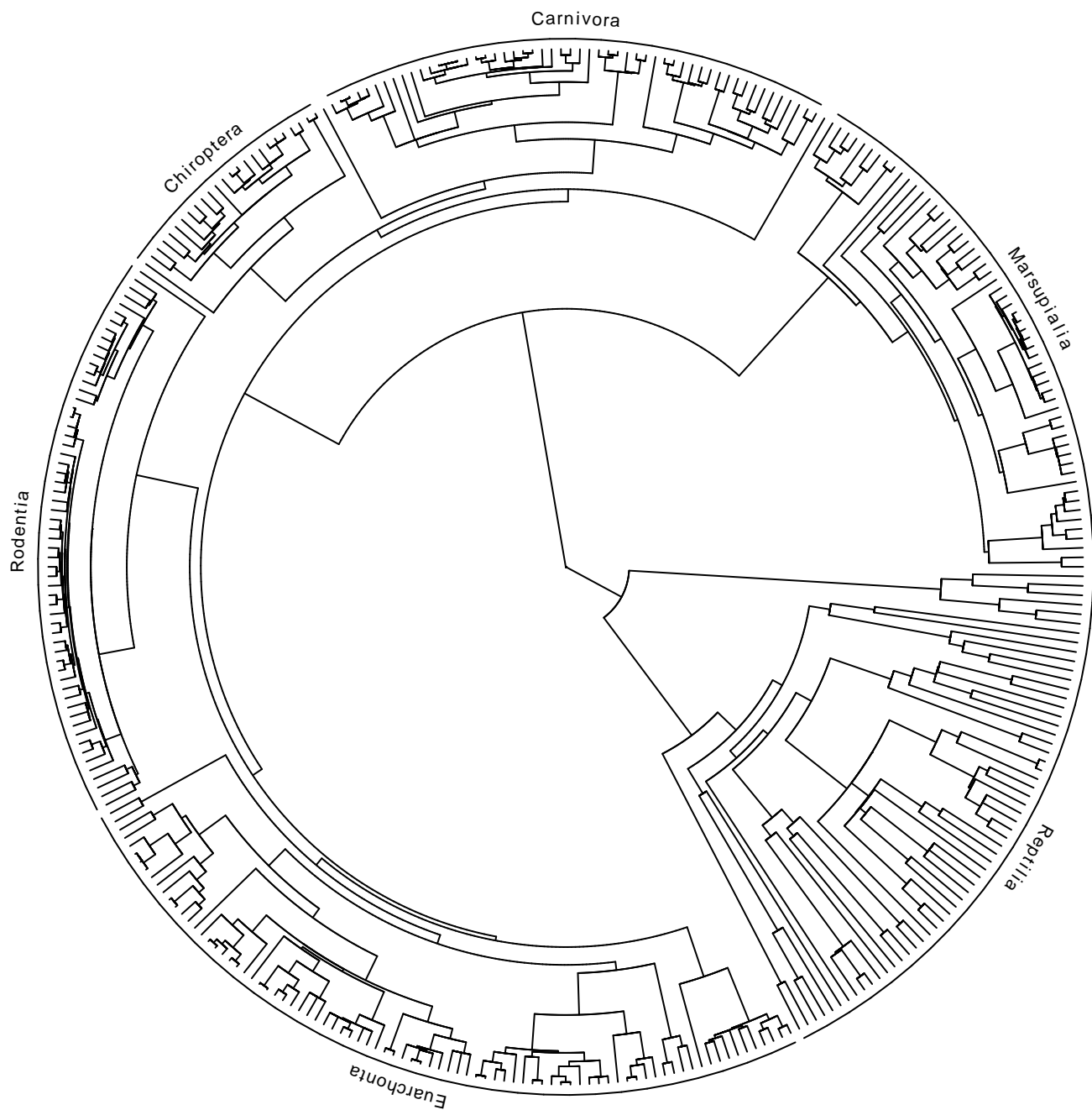

Supplement: S1 Fig — Made using TimeTree (Kumar et al. 2022). (PDF) [file pone.0292358.s004.pdf]

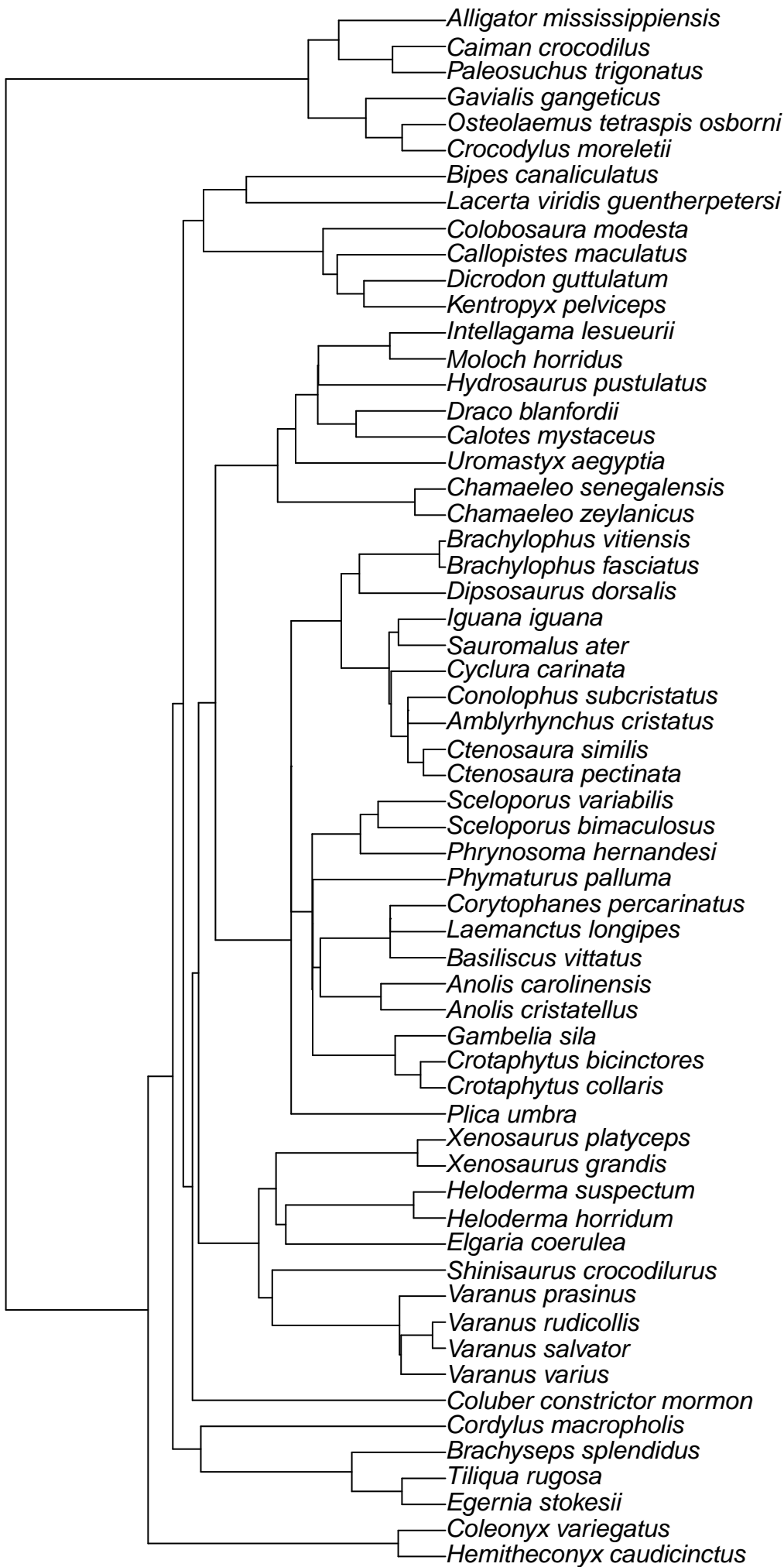

Supplement: S2 Fig — Made using TimeTree (Kumar et al. 2022). (PDF) [file pone.0292358.s005.pdf]

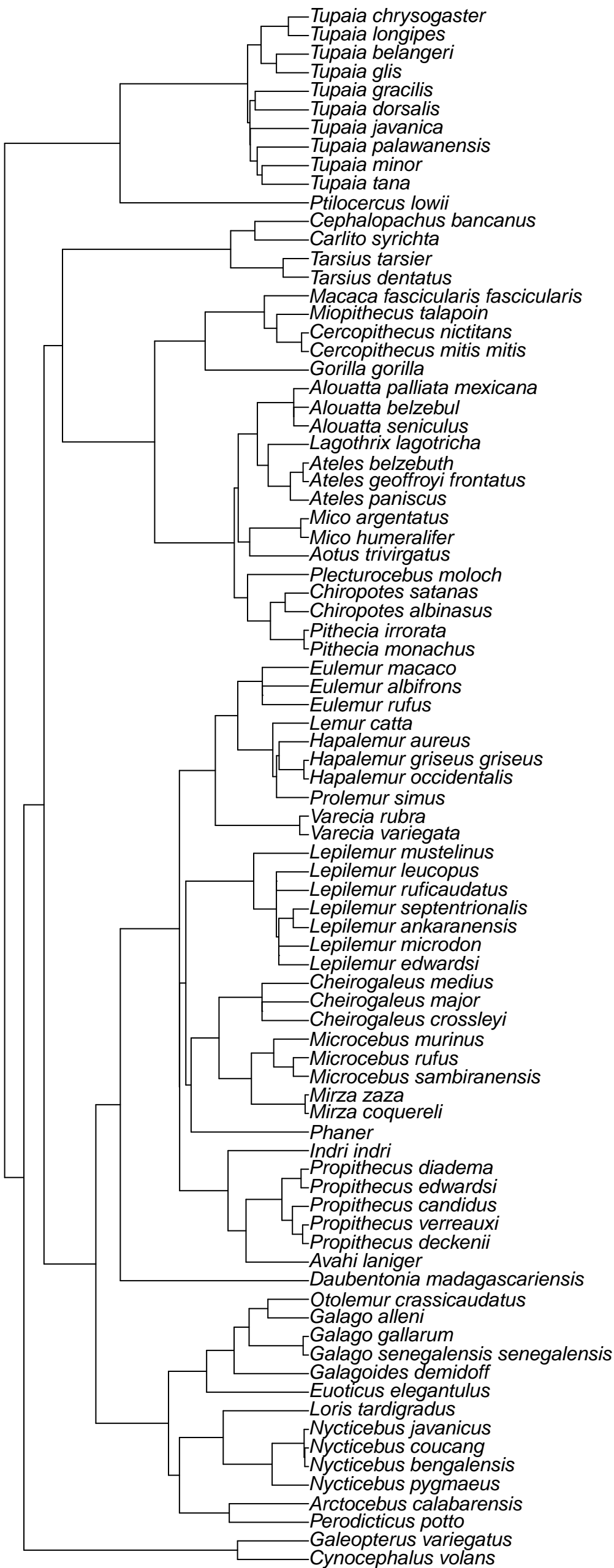

Supplement: S3 Fig — Made using TimeTree (Kumar et al. 2022). (PDF) [file pone.0292358.s006.pdf]

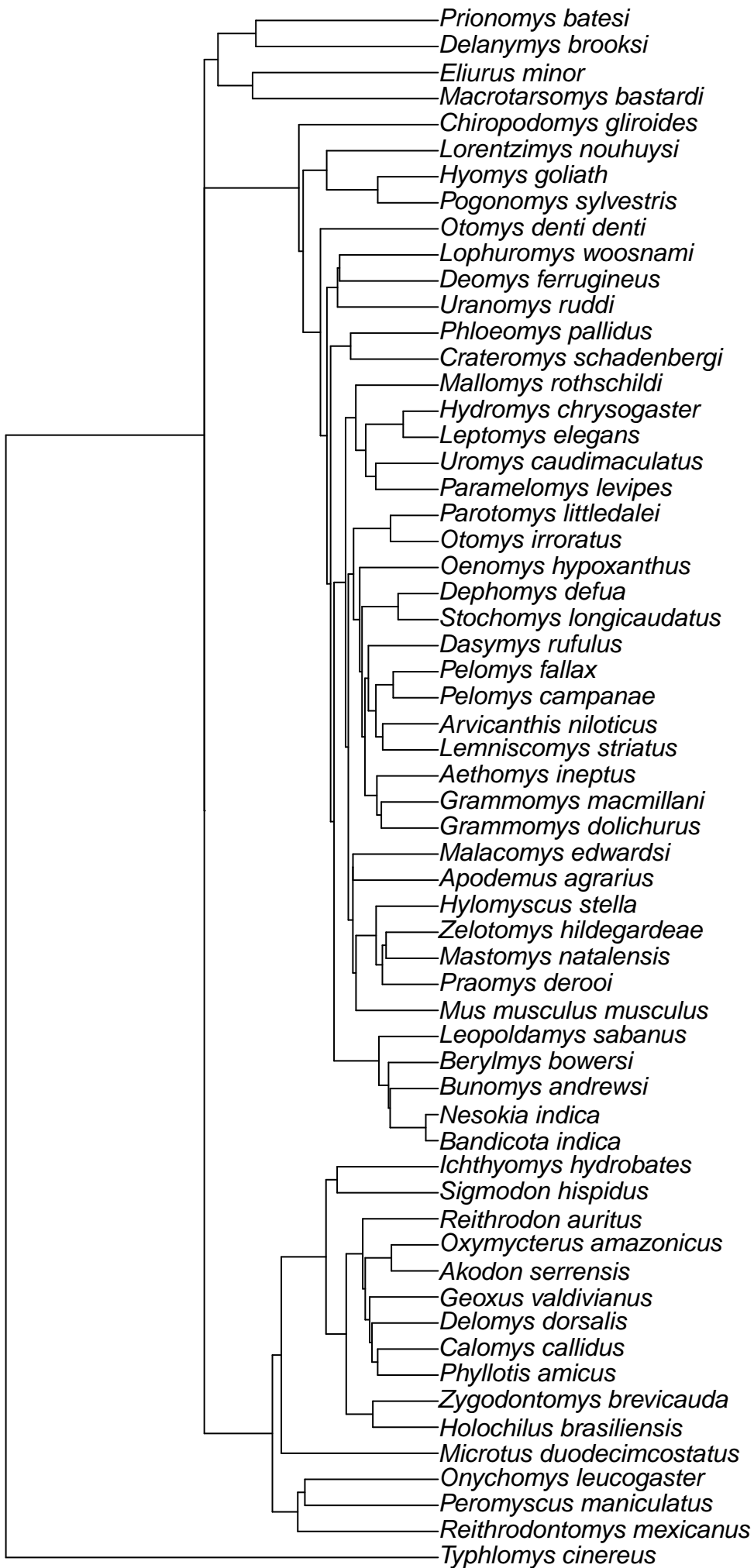

Supplement: S4 Fig — Made using TimeTree (Kumar et al. 2022). (PDF) [file pone.0292358.s007.pdf]

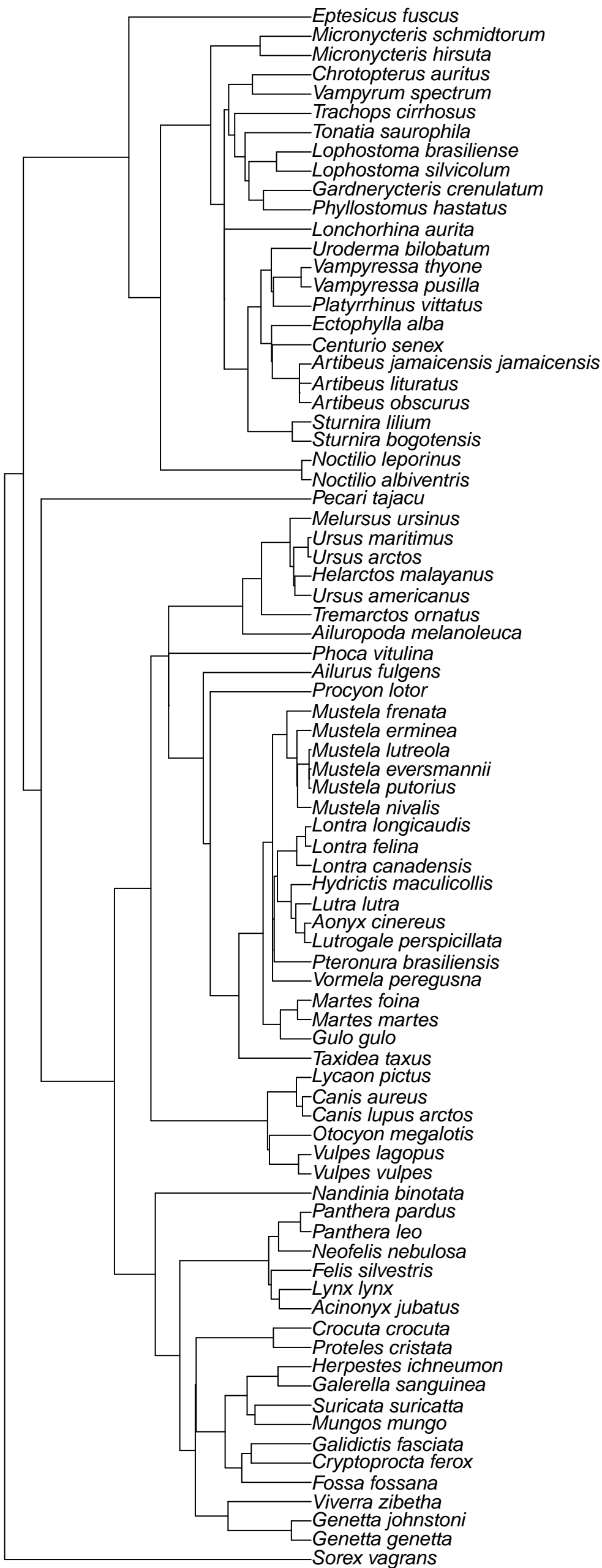

Supplement: S5 Fig — Made using TimeTree (Kumar et al. 2022). (PDF) [file pone.0292358.s008.pdf]

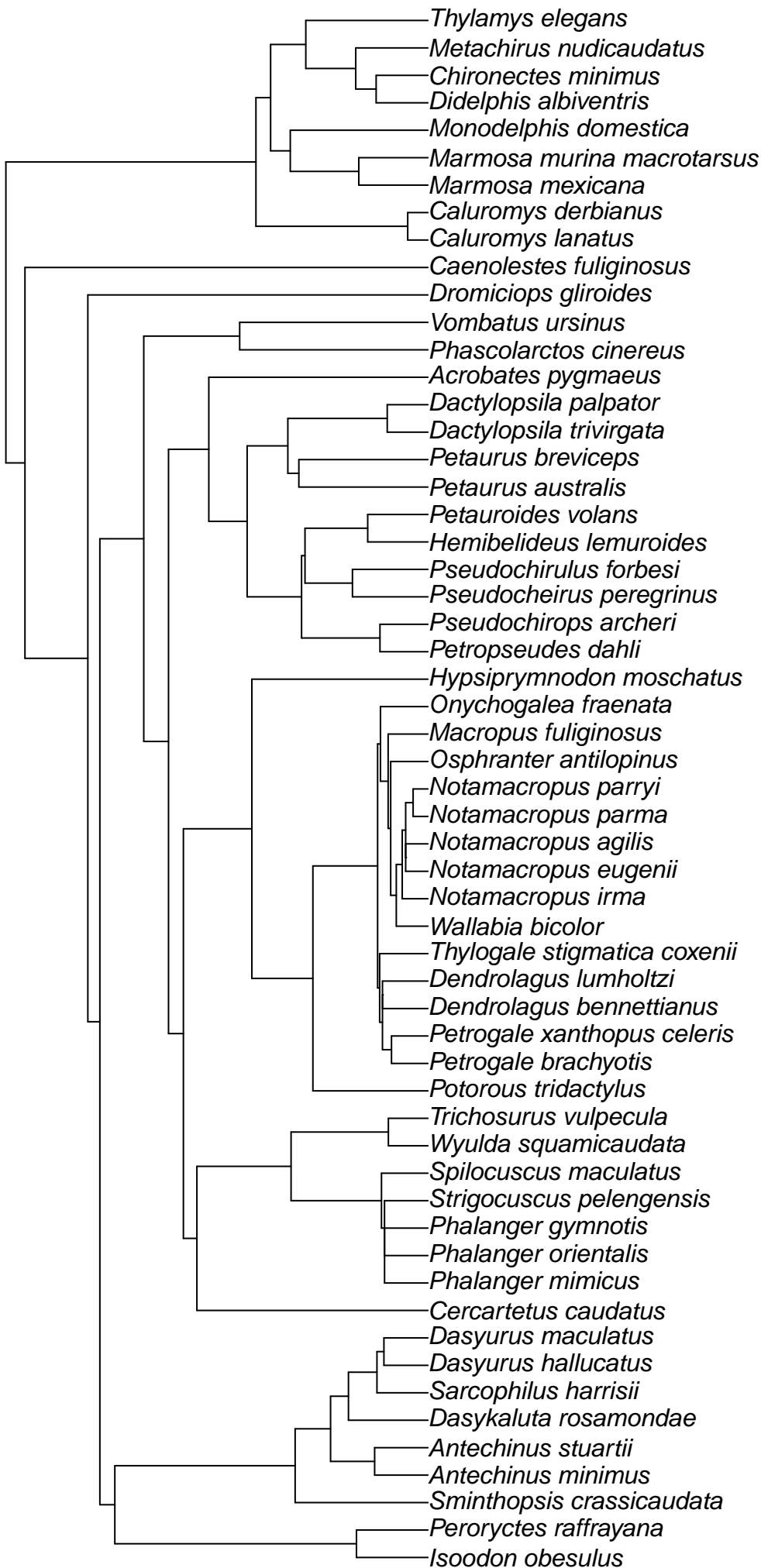

Supplement: S6 Fig — Made using TimeTree (Kumar et al. 2022). (PDF) [file pone.0292358.s009.pdf]
