# Supplementary material for: Dental complexity and diet in amniotes: A meta-analysis
Source: PLoS One. 2024 Feb 2;19(2):e0292358. doi: 10.1371/journal.pone.0292358 (PMC10836679; doi:10.1371/journal.pone.0292358)
Supplement: Attachment — Submitted filename: Response to Reviewers.pdf [file pone.0292358.s011.pdf]

THE OHIO STATE UNIVERSITY

Department of Evolution, Ecology, and Organismal Biology

300 Aronoff Laboratory
318 W. 12th Ave.
Columbus, OH 43210

614-292-8088 Phone
demers.29@osu.edu

August 31, 2023

To the Editors and Reviewers of **PLOS ONE:**

We are grateful to the feedback offered for improving our submitted manuscript. Based on this feedback, we incorporated several new analyses that strengthened our conclusions and revealed valuable new insights.

In response to the request for an analysis of phylogenetic signal, we calculated Blomberg's *K* for the dataset of one of the studies included in our meta-analysis. This analysis revealed that OPC(R) values are closely related to phylogeny and in some cases have a distribution that is not significantly different from a distribution due to a random walk along the phylogeny, supporting our overall claim about the importance of carefully considering the phylogenetic group of interest in OPC(R) analyses.

We repeated our analyses of variation among studies with a distinction between single- and multi-tooth studies, and were able to identify a trend for multi-tooth studies to find a greater difference in OPC(R) between herbivores and faunivores.

We followed the suggestion to emphasize the importance of differing methods among studies of OPC(R) by including a new table (Table 1) and reiterating this point in the text in both the Introduction and the Discussion.

Existing figures and tables were edited in accordance with the reviewer requests.

We appreciate the reviewer inquiries about our stance in regard to how phylogenetic signal has been dealt with previously. We agree that in most published studies phylogeny is properly accounted for either in the choice of sample or in formal analyses. We have clarified our manuscript to make more apparent that our concern is with comparing OPC(R) values across distantly related taxa, as is often done with fossils, and not with how phylogenetic signal is handled within most individual studies.

We also made the following changes, at the line numbers specified:

- Wording was changed to clarify the difference in digestability between plant and animal matter (61).
- The importance of differing methodologies was emphasized in the text (92).
- A table was added to highlight the varied and changing methods used in OPC(R) analysis (112).
- A table was added to clarify the diets seen in the included studies and how they were categorized here (182).
- The two forest diagrams were relocated in the manuscript to be more relevant to the text (317, 323).

THE OHIO STATE UNIVERSITY

Department of Evolution, Ecology, and Organismal Biology

300 Aronoff Laboratory
318 W. 12th Ave.
Columbus, OH 43210

614-292-8088 Phone
demers.29@osu.edu

- Notes were added on intraspecific variation in OPC(R) seen in other studies (367).
- Table 4 was altered so that differences are now expressed as a correct "Percentage difference" (374).
- Additional advice was added on using OPC(R) given the variation in methods currently seen and strength of phylogenetic signal (428).
- Figures were changed to add clarity and distinguish single- from multi-tooth studies.
- The financial disclosure and funding information for the paper were brought into agreement.
- The manuscript and other submitted files were reformatted to match journal standards.
- Minor wording was changed throughout for readability.
- References were checked for retractions; no retractions were found.
- Three references were added: [68] Blomberg et al. 2003, [69] Revell et al 2012, and [71] Pampush et al. 2016

We thank you again for your feedback.

Sincerely,

Anessa DeMers, Ph.D.
Department of Evolution, Ecology, & Organismal Biology
