# Supplementary material for: Dental complexity and diet in amniotes: A meta-analysis
Source: PLoS One. 2024 Feb 2;19(2):e0292358. doi: 10.1371/journal.pone.0292358 (PMC10836679; doi:10.1371/journal.pone.0292358)
Supplement: Attachment — Submitted filename: Reviewer comments - Christison.docx [file pone.0292358.s010.docx]

Reviewer comments

This study asks an important question that will have implications for any analyses using OPC or OPCR. This is good, rigorous work, and I recognize that compiling and testing data collected in previous studies can be met with backlash from those involved in the original data collection. I want therefore to thank the authors for doing this study that will allow researchers moving forward to make informed decisions about which methods to use in their analyses.

There are two main topics I want to touch on related to this study: the first is the claim that OPC/R analyses are often done without any regard for phylogeny or phylogenetic signal. Of the 16 studies cited in Table 1, four appear to have no regard for phylogeny (cited as 46, 9, 51, and 10), and three account for phylogeny by only looking at crown taxa (e.g. bears (50), primates (29, 45), rodents (36)) as you suggest. All of the other studies take it into account either through statistical analyses (ANOVA, Mann-Whitney U test, pairwise phylogenetically informed regression analyses), or through their interpretation of their results (suggesting topographical analyses should be paired with phylogenetic analyses). It is well-established that phylogenetic signals are extremely strong in mammalian teeth, and so the claim that researchers have been conducting morphological studies without accounting for phylogeny must be backed up. Is their accounting for phylogeny insufficient? Can you demonstrate that?

The second thing I want to touch on is the issue of methodology between studies. Several times you mention that the methodology to determine OPC/R is variable between studies. This effectively means that researchers are each following their own methodologies and that these data cannot be comparable. This should be made quite clear; if people are not standardising their dental meshes to 10 000 polygons, or are deviating from the original methods set out in Evans et al. 2007, then their data should absolutely not be combined.

Brigid E. Christison

64-68: It’s not just the tough cell structure of plants that causes differences in processing methods. Yes, vertebrate flesh is easier to break down, but it must be sliced with highly specialized dentition. A brief comment on this is warranted.

82: be specific when talking about diets. OPC and OPCR are used to infer the average diets that an animal has adapted to eat, as opposed to the specific diet of an individual.

87: I did this too! Christison et al. 2022.

88: I’d say “haphazardly” is unfair in this instance. Using published data is a very established method of conducting science, and before the present study it was a fair assumption that they would be transferrable.

92-93: see note about phylogeny

95: Reiterate the assumption at the beginning of the paragraph

96-97: the statement that “variation of these methods has been found to affect results” is vital to this study. If the data was collected by different methods, then they are not comparable. A more damning statement here would be to point out that researchers are varying in the way that they follow the methods, this is effectively new information since one would hope we were all following the methods correctly. More on this later (lines 324-332).

99-100: The statement that distinct dietary categories obscure dietary variation is correct; however, an explanation as to why people even use the categories and what they are based on is warranted. Typically, it is for analytical purposes, and again meant to reflect the average diet an animal has adapted to rather than the diet of the individual. Pineda-Munoz et al. 2017 reports how they established their categories based on stomach contents, for example. I’ve also used Elton Traits 1.0 (Wilman 2014), a database of species-level info from field guides, and dietary categories from Evans et al. 2007 and Van Valkenburgh et al. 2004.

Stable dietary categories are meant to work as general guidelines based on the average diets that an animal has adapted to eat. I am not defending them nor advocating them but merely explaining the reason they are used and can be useful especially when looking at extinct animals whose diets and ecologies are poorly-understood.

106-109: note on phylogeny

124: style preference: it can be useful to re-define acronyms the first time they are used in each section

169-174: For data extraction and comparing different dietary categories to each other, I wonder if it would be possible to standardize dietary categories. Studies **should** theoretically explain how they determined their dietary categories, and possibly lead to the data they used. For example if study A separated frugivores and folivores using a database of stomach contents, and study B only used a category of “herbivore” using a database of observed diets, it may be possible to look at the databases directly to determine dietary categories. If you’re able to eliminate the different ways diets are categorized as a variable it could strengthen your study.

251: phylogeny!

294-296: Nailing down the differences in methodology is vital to determining if these studies are comparable or not. I’d also like some clarification on OPC/R for tooth row vs individual teeth, because these data are not comparable. I know Pineda-Munoz et al 2017 uses average OPCR (OPCR divided by number of teeth in the tooth row) which would be more comparable to individual teeth, but still not perfect. It may be worth standardising all the data here to mean OPC/R if you want to ensure they are comparable.

You should also specify *which* individual teeth are being looked at; for example in carnivorans it is established that the carnassial teeth (m1 and P4) contain the most information about an animal’s diet, while some teeth may contain little to no dietary info. If individual teeth are being looked at but they are different teeth in the tooth row, this may significantly impact your results.

325-332: “similar enough methods” is unclear; how do they differ? Differences in methodology are crucial to figuring out if these data are comparable.

394: It is worth noting that thus far, no studies have examined the impact of mesowear on OPC/R values within individual species.

Citations

Christison, Brigid E., F. Gaidies, S. Pineda-Munoz, A. Evans, and D. Fraser. Dietary niches of creodonts and carnivorans of the late Eocene Cypress Hills Formation. Journal of Mammalogy 103: 2–17.

Evans A.R., Wilson G.P., Fortelius M., Jernvall J. 2007. High-level similarity of dentitions in carnivorans and rodents. Nature 445:78–81.

Pineda-Munoz S., Lazagabaster I.A., Alroy J., Evans A.R. 2017. Inferring diet from dental morphology in terrestrial mammals. Methods in Ecology and Evolution 8:481–491.

Van Valkenburgh B., Wang X., Damuth J. 2004. Cope’s rule, hypercarnivory, and extinction in North American canids. Science (New York, N.Y.) 306:101–104.

Wilman H., Belmaker J., Simpson J., de La Rosa C., Rivadeneira M., Jetz W. 2014. EltonTraits 1.0: species-level foraging attributes of the world’s birds and mammals. Ecology 95:2027.
